# Supplementary material for: The Pick fold in tau filaments from human MAPT mutants
Source: Acta Neuropathol. 2026 Jul 8;152(1):6. doi: 10.1007/s00401-026-03049-8 (PMC13346262; doi:10.1007/s00401-026-03049-8)
Supplement: Supplementary file 1 — Supplementary file1 (PDF 14227 KB) [file 401_2026_3049_MOESM1_ESM.pdf]

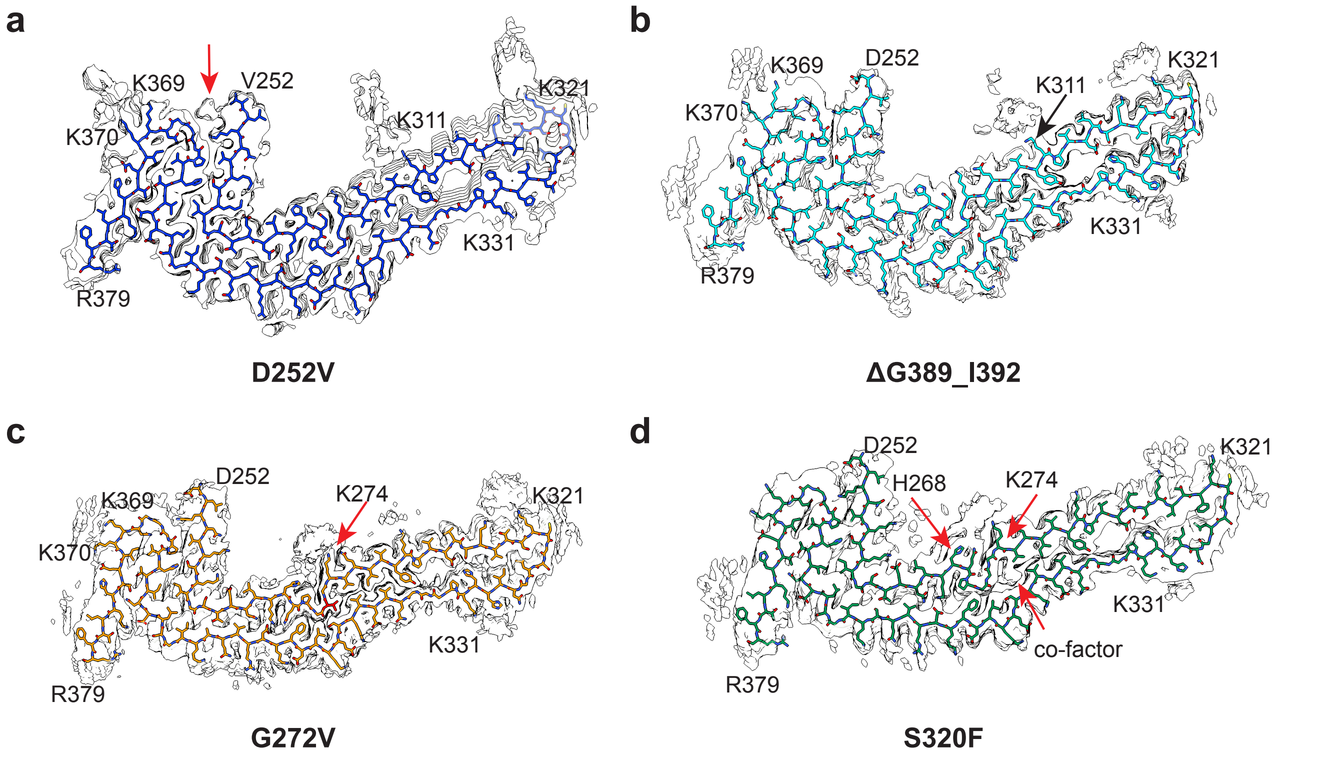

Figure S1: Cryo-EM maps of tau filaments from individuals with *MAPT* mutations D252V, ΔG389_I392, G272V and S320F, showing at low contour levels to reveal additional densities on the filament surface.

a, Cryo-EM map of D252V tau filaments. The red arrow indicates the additional density near residue V252.

b, Cryo-EM map of ΔG389_I392 tau filaments.

c, Cryo-EM map of G272V tau filaments. The red arrow indicates the additional density near residue K274.

d, Cryo-EM map of S320F tau filaments, the red arrows indicate the additional densities near residue G272 and residues H268 and K274.

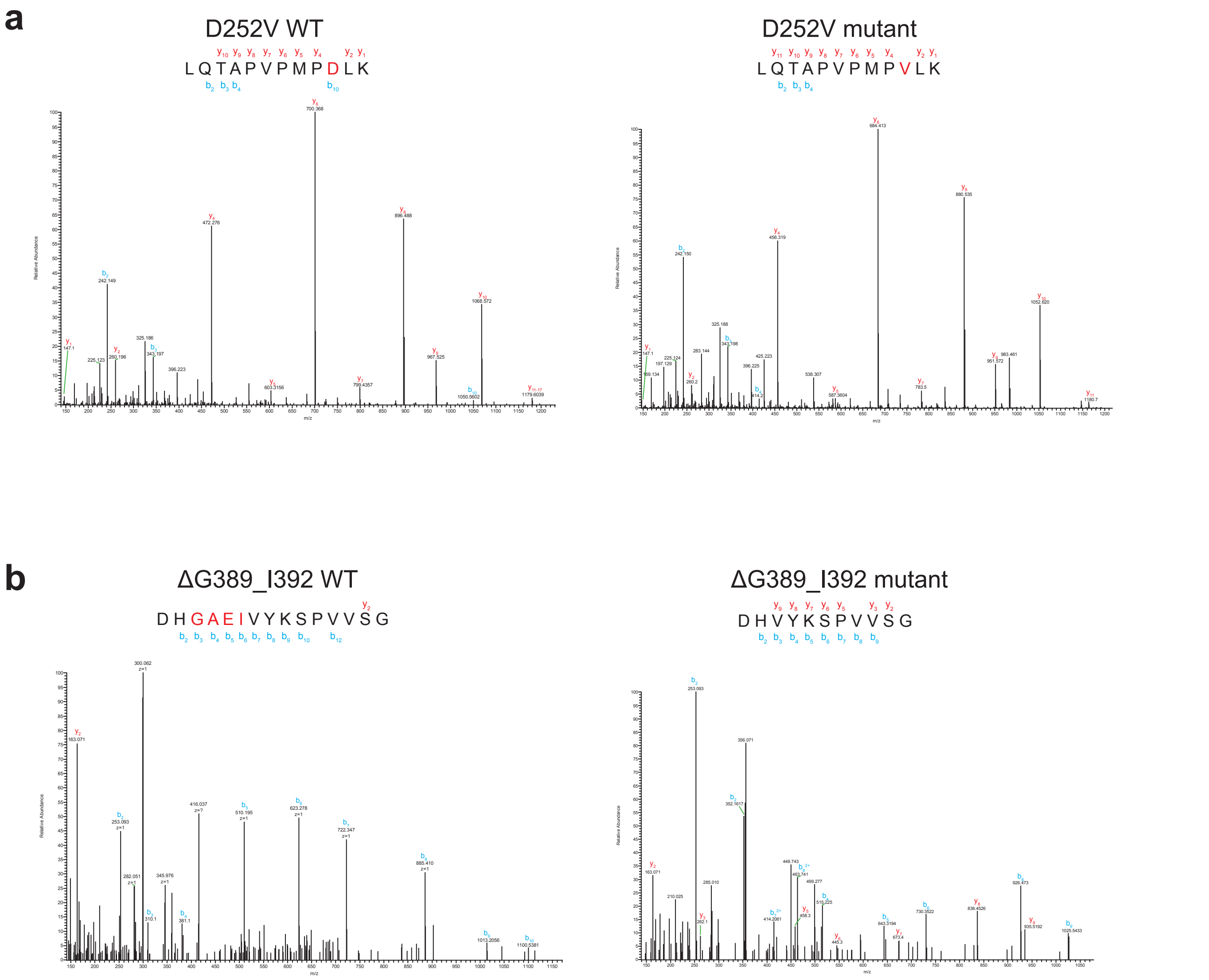

Figure S2: Mass spectrometric analysis of tau from the sarkosyl-insoluble fractions of cases with *MAPT* mutations encoding D252V tau (frontal cortex) and ΔG389_I392 tau (frontal cortex).

a, MSMS mass spectra of sarkosyl-insoluble tau from the D252V case. **b,** MALDI mass spectrum of sarkosyl-insoluble tau from the ΔG389_I392 case. In both cases, peptides corresponding to wildtype (WT) and mutant tau were detected.

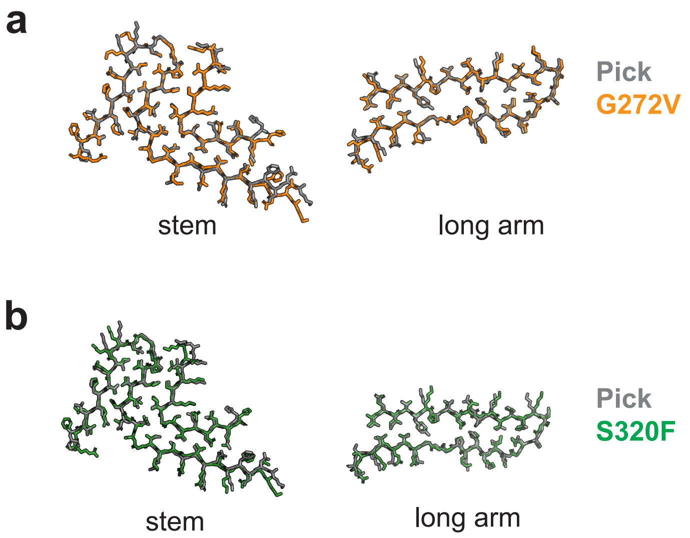

Figure S3: Comparison of G272V and S320F tau filaments with those from sporadic Pick's disease.
a, Overlay of structures with the G272V mutation (orange) with the Pick fold (grey).
b, Overlay of structures with the S320F mutation (green) with the Pick fold (grey).

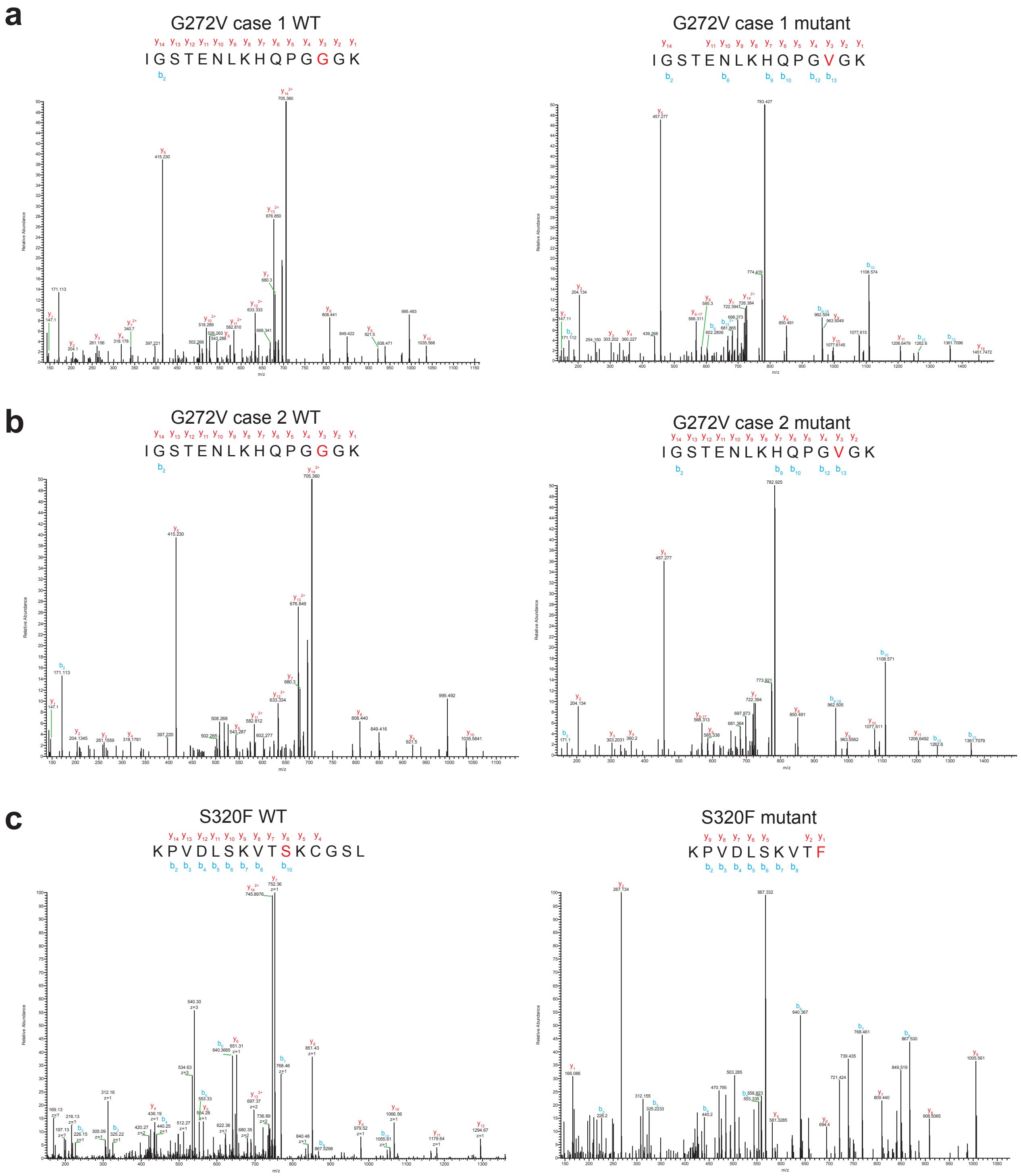

Figure S4: Mass spectrometric analysis of tau from the sarkosyl-insoluble fractions of cases with *MAPT* mutations encoding G272V tau (temporal cortex) and S320F tau (caudate nucleus).

a, MSMS mass spectra of tau filaments extracted from G272V case 1. **b,** MSMS mass spectra of tau filaments extracted from G272V case 2. **c,** MSMS mass spectra of tau filaments extracted from the S320F case. Peptides corresponding to both wildtype (WT) and mutant tau were detected.

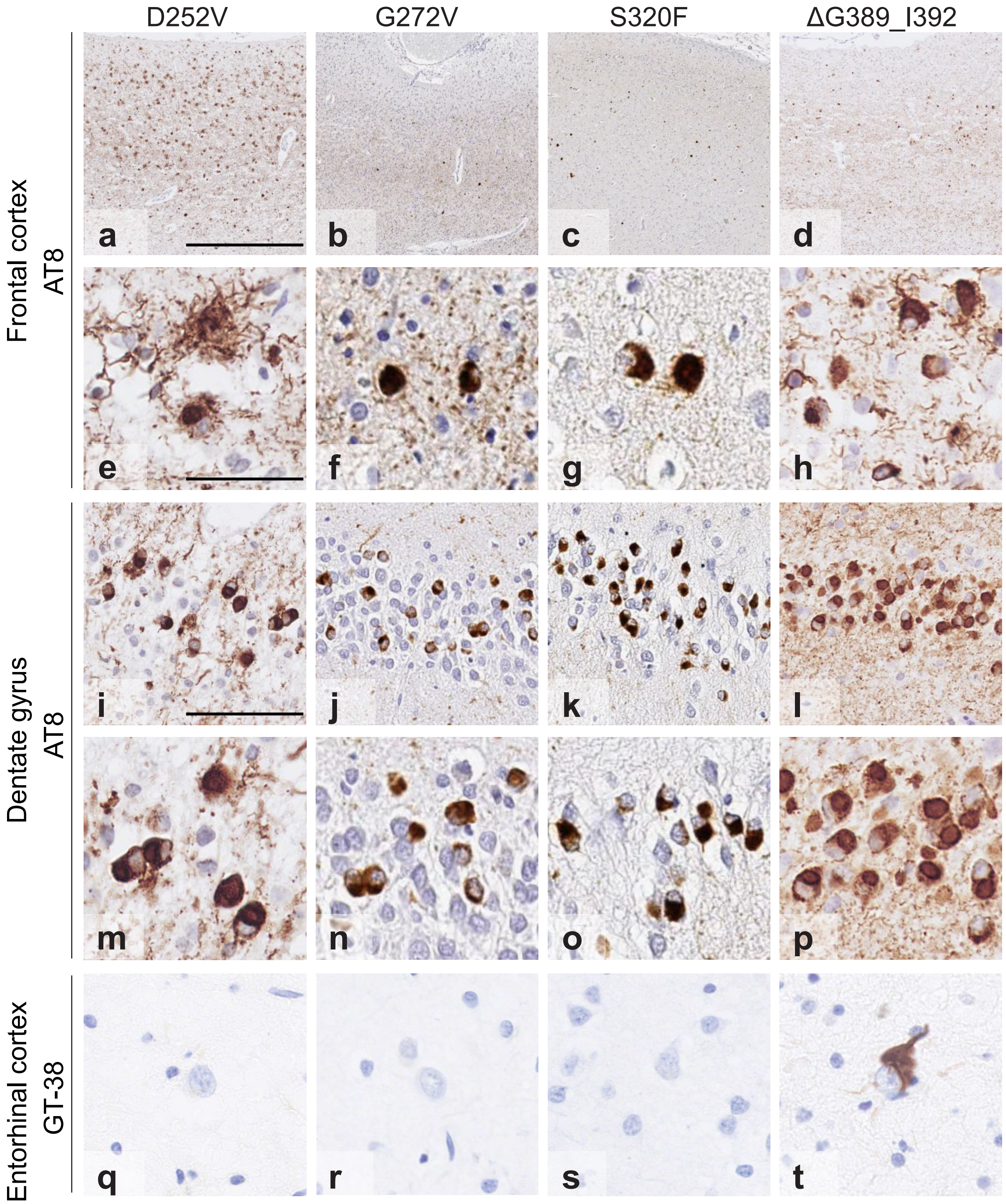

Figure S5: Tau inclusions in frontal cortex, dentate gyrus and entorhinal cortex from individuals with *MAPT* mutations encoding D252V, G272V, S320F and ΔG389-I392 tau.

a-h, AT8 immunohistochemistry of the frontal cortex; **i-p,** AT8 immunohistochemistry of the dentate gyrus; **q-t,** GT-38 immunohistochemistry of the entorhinal cortex. Scale bars: 600 µm in a (for a-d); 50 µm in e (for e-h, m-t); 100 µm in i (for i-l).

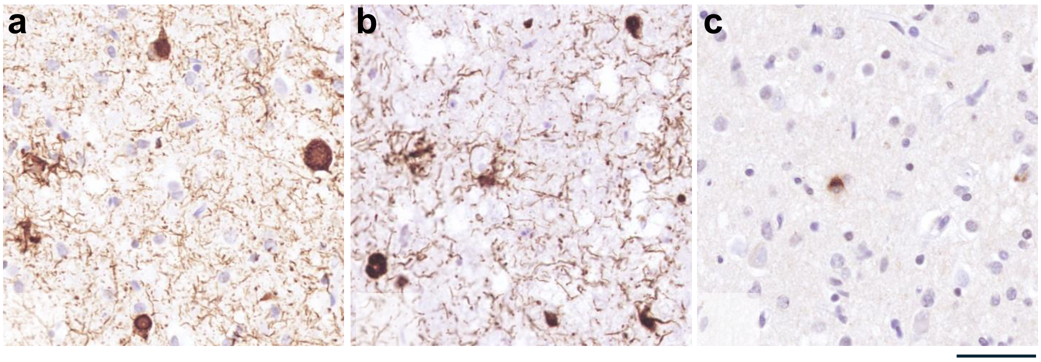

Figure S6: Tau pathology in the frontal cortex of the individual with *MAPT* mutation ΔG389-I392.

a, AT8 immunohistochemistry shows a dense meshwork of tau-positive neuropil threads, together with abundant neuronal cytoplasmic inclusions showing tangle, pre-tangle and Pick body-shaped morphologies, as well as astrocytic tau pathology consisting of ramified astrocytes. **b,** 3R tau immunostaining shows similar morphologies and distributions as for AT8, confirming the predominance of 3R tau-positive neuronal and glial inclusions. **c,** 4R tau immunostaining shows rare neuronal cytoplasmic inclusions. Scale bar: 50 μm.

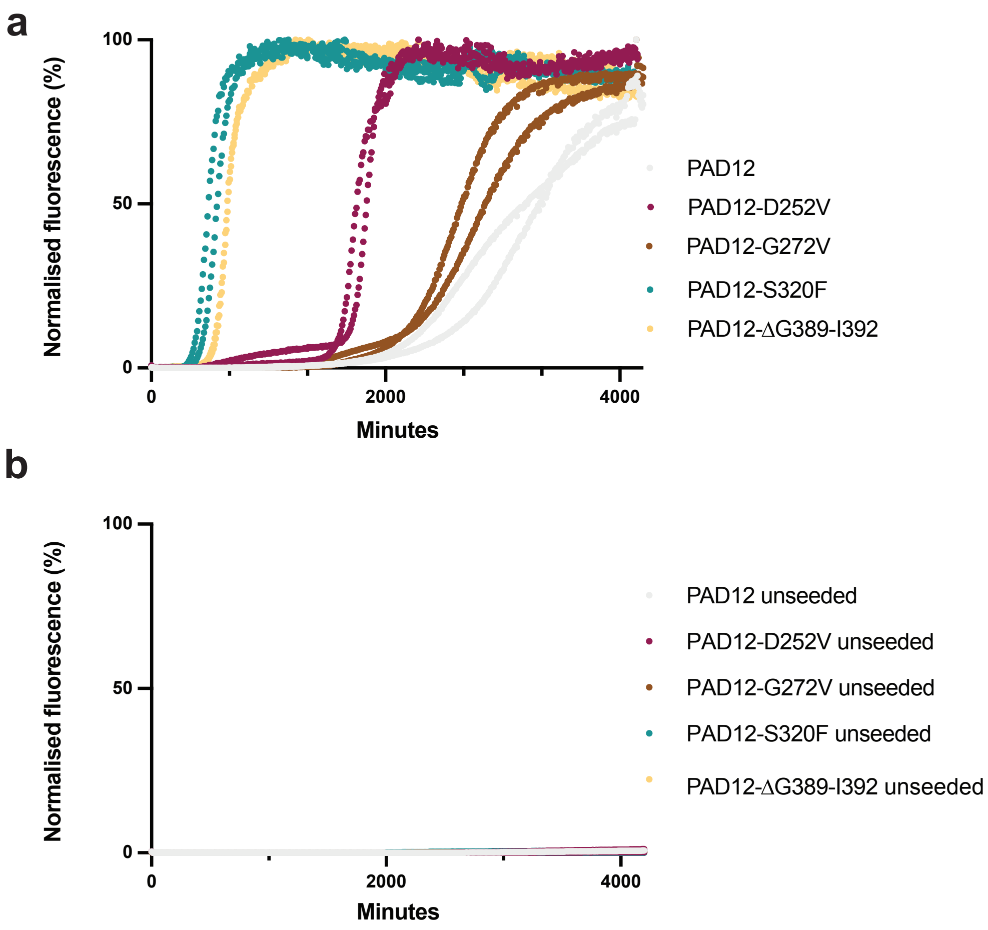

Figure S7: ***In vitro*** **assembly of PAD12 tau and PAD12 tau with FTDP-17T mutations encoding D252V, G272V, S320F and ΔG389-I392 tau monitored by thioflavin T fluorescence.**
a, Seeded assembly.
b, Unseeded assembly.

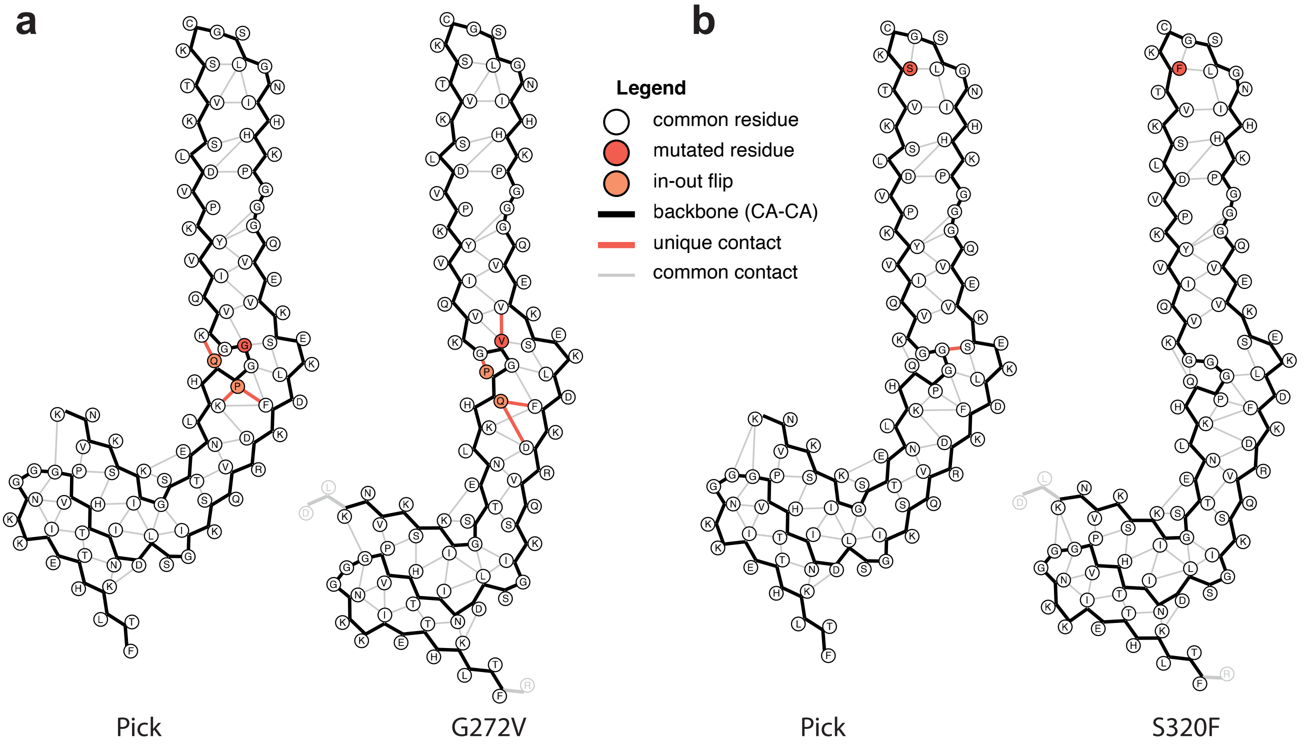

Figure S8: Amyloid packing difference comparisons.
a, Comparison between the Pick fold and the G272V fold. **b,** Comparison between the Pick fold and the S320F fold.
Side chain packing interactions (i.e. residue pairs with any side chain atoms, including Cα atoms for glycines, below 4.5 Å from each other) are shown in grey lines if they exist in both folds and in dark orange lines if they only exist in one of the folds. Mutated residues are highlighted in red circles; residues that have a different side chain orientation, i.e. facing either to the inside or the outside of the ordered core, are highlighted in orange circles. The amyloid packing difference (APD), a new metric for the quantitative comparison of amyloid folds (40), which is calculated as the percentage of residues that are unique in the largest fold that are engaged in distinct unique contacts in one of the folds, or that have a different inside-out orientation between the two folds, is 12% for the Pick-G272V and 5% for the Pick-S320F comparisons.

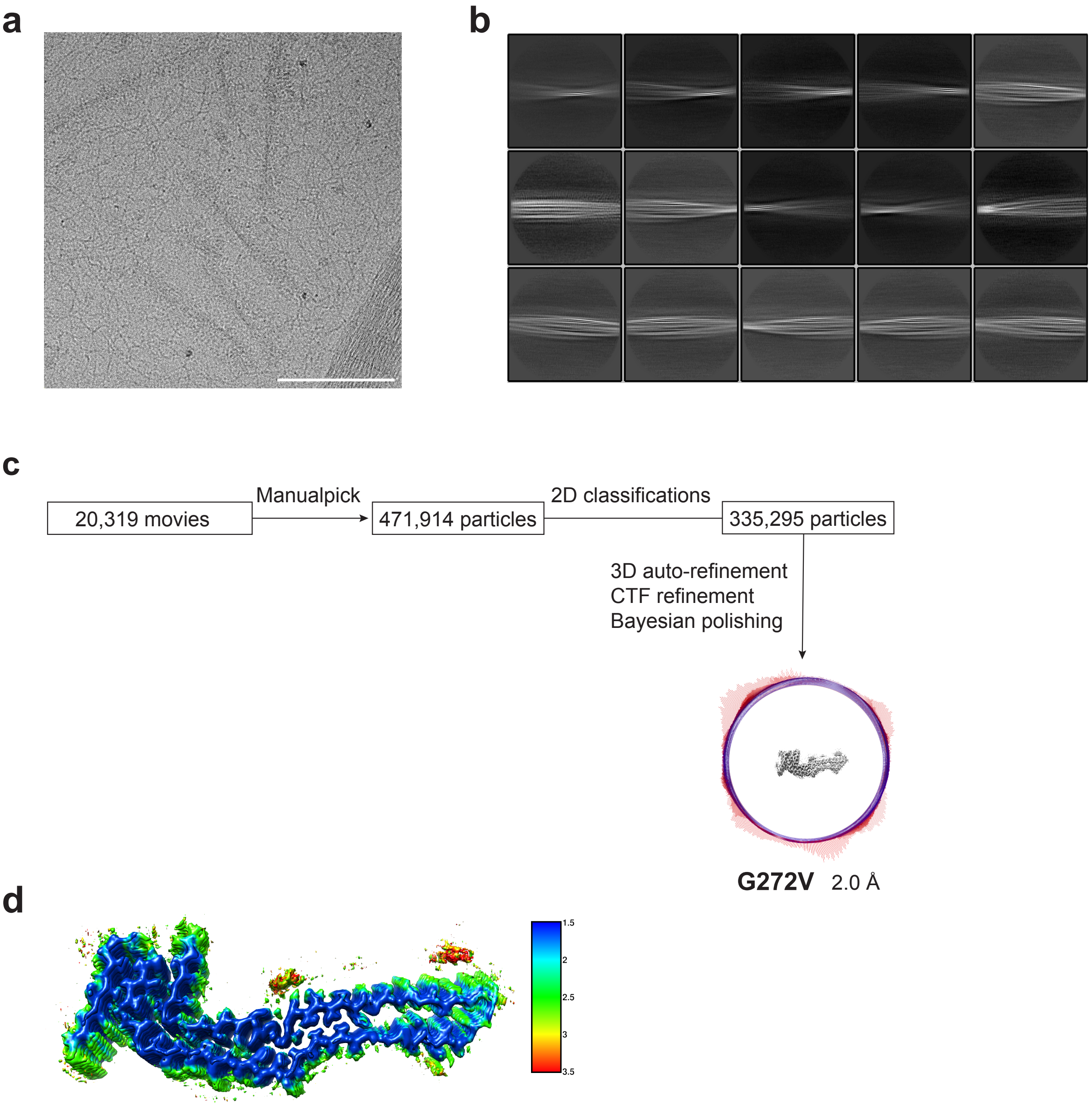

Figure S9: Cryo-EM image processing workflow for the G272V tau filaments.
a, Representative motion-corrected micrograph. Scale bar, 100 nm. **b,** Representative 2D class averages; the box size is 762 Å. **c,** Workflow of cryo-EM imaging. The angular distribution histogram of the final reconstruction is shown at the bottom. **d,** Local resolution maps of G272V tau estimated using RELION.

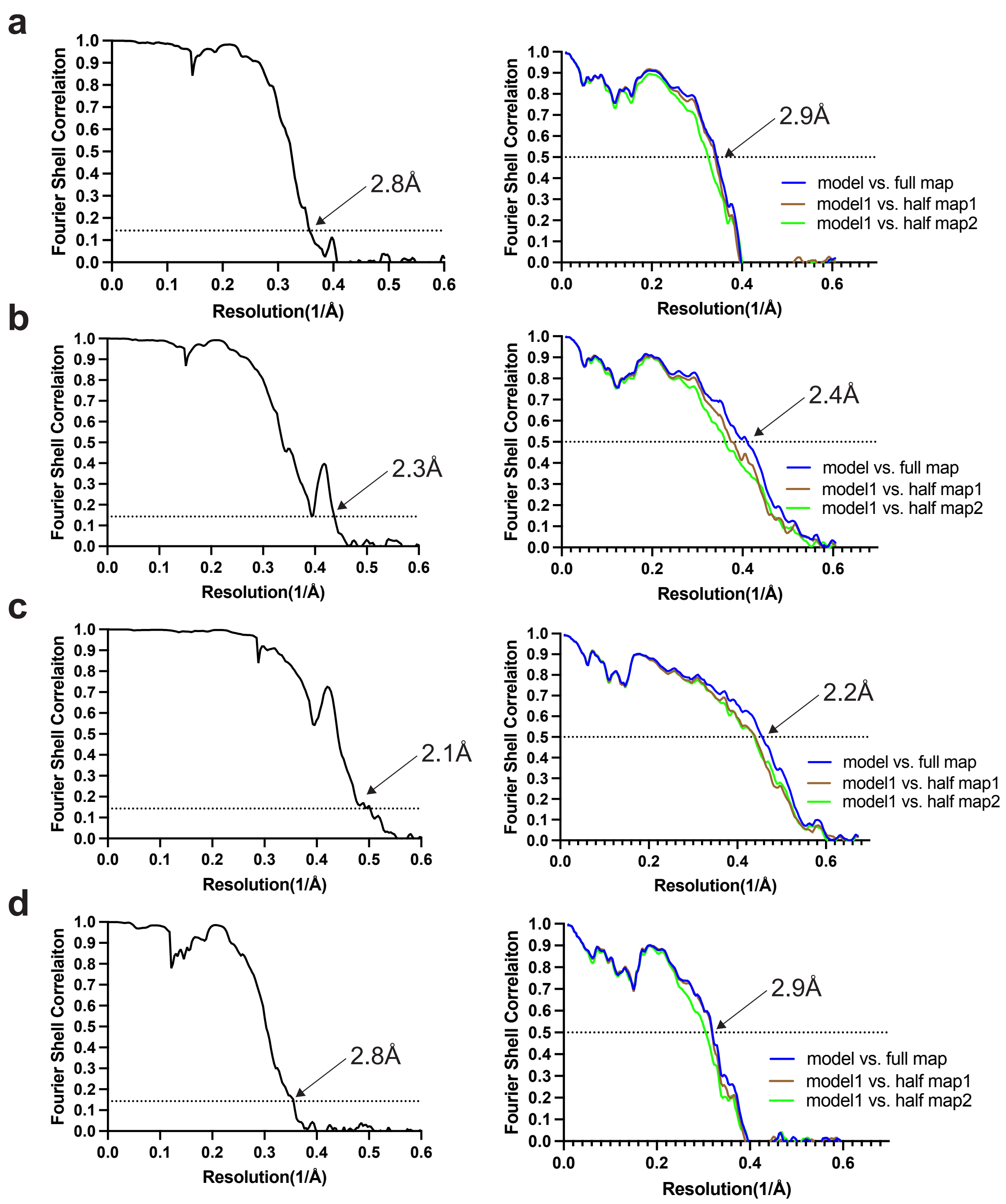

Figure S10: Fourier shell correlation (FSC) curves for cryo-EM maps (left panel) and model to map validation (right panel).
a, D252V tau structure.
b, ΔG389_I392 tau structure.
c, G272V tau structure.
d, S320F tau structure.

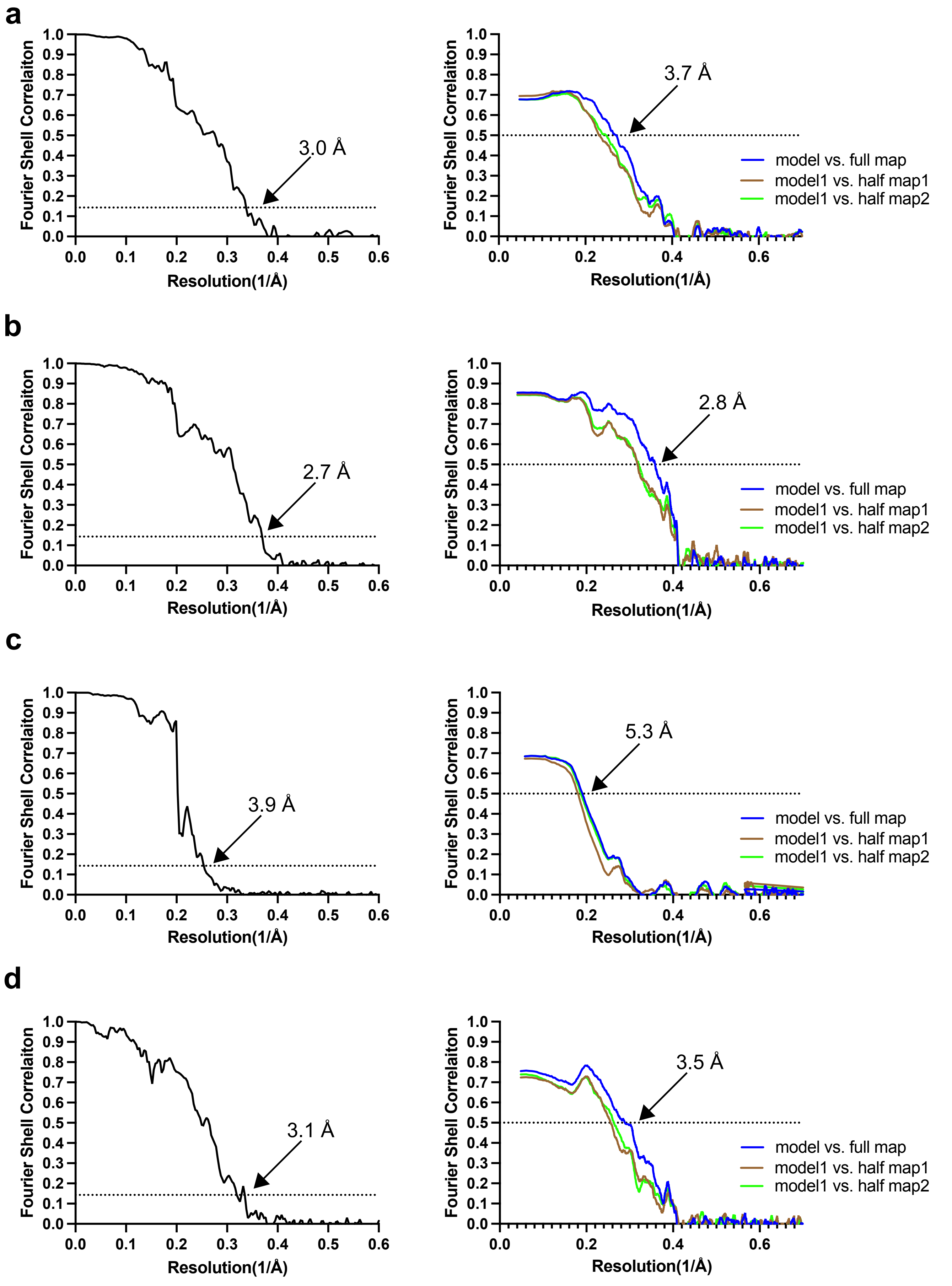

Figure S11: Fourier shell correlation (FSC) curves for cryo-EM maps (left panel) and model to map validation (right panel).
a, Recombinant D252V tau singlet. **b,** Recombinant D252V tau doublet. **c,** Recombinant G272V tau singlet. **d,** Recombinant G272V tau doublet.

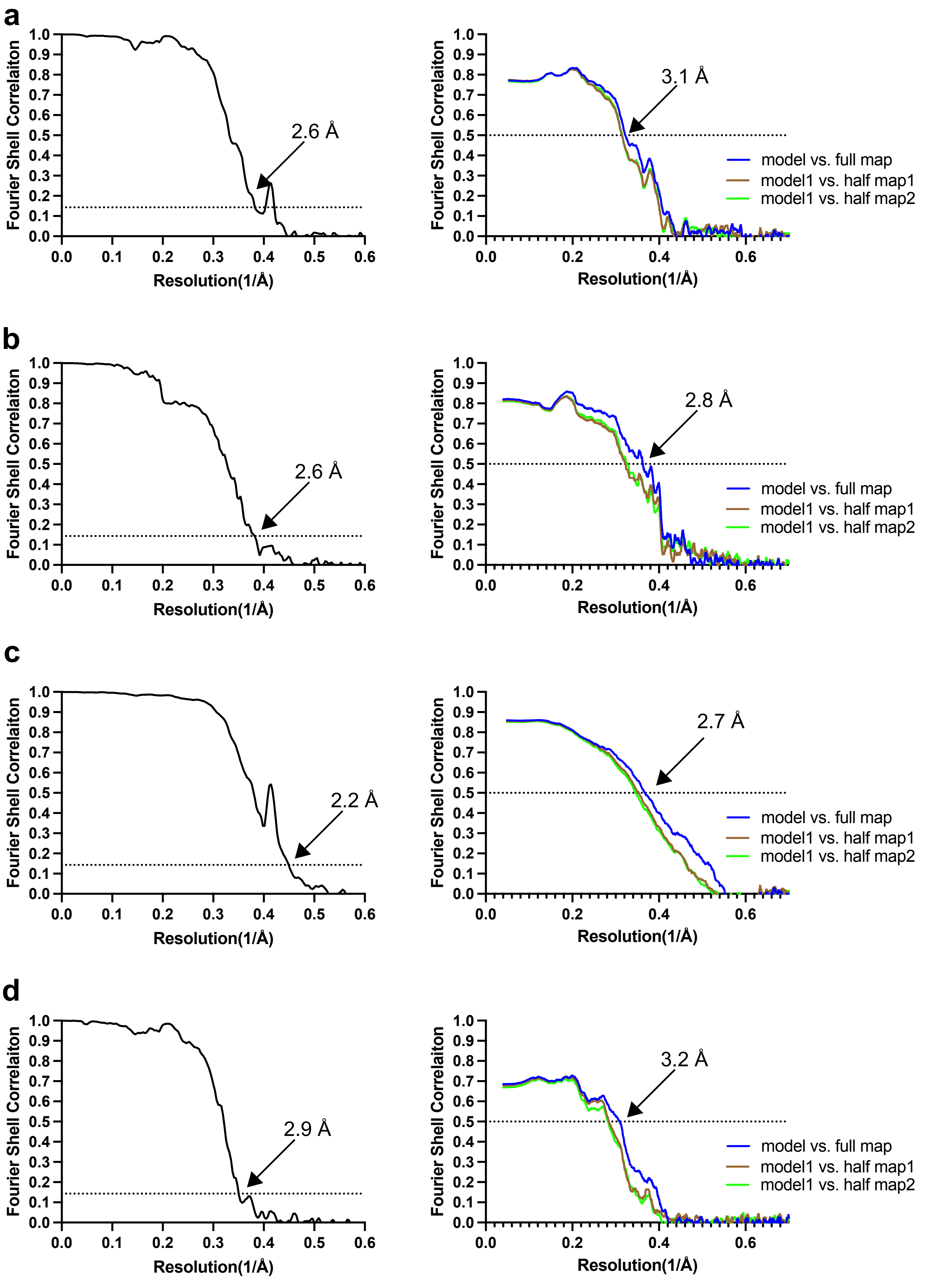

Figure S12: Fourier shell correlation (FSC) curves for cryo-EM maps (left panel) and model to map validation (right panel).
a, Recombinant S320F tau Pick singlet. **b,** Recombinant S320F tau Pick doublet. **c,** Recombinant S320F tau type 2 singlet. **d,** Recombinant S320F tau type 2 doublet.
