# Supplementary material for: The Pick fold in tau filaments from human MAPT mutants
Source: Acta Neuropathol. 2026 Jul 8;152(1):6. doi: 10.1007/s00401-026-03049-8 (PMC13346262; doi:10.1007/s00401-026-03049-8)
Supplement: Supplementary file 2 — Supplementary file2 (DOCX 27 KB) [file 401_2026_3049_MOESM2_ESM.docx]

Table S1: Cryo-EM data collection, refinement and validation statistics

|  | D252V | ΔG389_I392 | G272V  Case 2 | S320F |
| --- | --- | --- | --- | --- |
| **Data collection** |  | | | |
| Microscope | Titan Krios | | | |
| Voltage (kV) | 300 | | | |
| Detector | Falcon4i | K3 | Falcon4i | Falcon4i |
| Electron exposure (e–/Å^2^) | 40 | 40 | 40 | 40 |
| Defocus range (μm) | -1.0 to -2.0 | -1.0 to -2.0 | -1.0 to -2.0 | -1.0 to -2.0 |
| Pixel size (Å) | 0.824 | 0.826 | 0.744 | 0.826 |
| **Data processing** |  |  |  |  |
| Box size (pixel) | 400 | 400 | 400 | 400 |
| Symmetry imposed | C1 | C1 | C1 | C1 |
| Initial particle images (no.) | 196,865 | 321,528 | 471,914 | 331,114 |
| Final particle images (no.) | 124,055 | 143,931 | 335,295 | 40,748 |
| Map resolution (Å)  FSC threshold 0.143 | 2.8 | 2.3 | 2.1 | 2.8 |
| Helical rise (Å) | 4.83 | 4.80 | 4.89 | 4.86 |
| Helical twist (°) | -0.7 | -0.73 | -0.66 | -0.65 |
| **Refinement** |  |  |  |  |
| Model resolution (Å)  FSC threshold 0.5 | 2.9 | 2.4 | 2.2 | 2.9 |
| Map sharpening *B* factor (Å^2^) | -40 | -34 | -27 | -39 |
| Model composition  Non-hydrogen atoms  Protein residues  Ligands | 2948  388  0 | 3690  485  0 | 2223  291  0 | 2972  388  0 |
| *B* factors (Å^2^)  Protein | 52.9 | 48.1 | 41.4 | 52.8 |
| R.m.s. deviations  Bond lengths (Å)  Bond angles (°) | 0.0084  1.983 | 0.0048  1.438 | 0.0049  1.371 | 0.0085  1.797 |
| Validation  MolProbity score  Clashscore  Poor rotamers (%) | 1.88  3.8  3.57 | 1.87  5.43  2.38 | 1.57  1.32  3.53 | 2.65  8.23  8.33 |
| Ramachandran plot  Favoured (%)  Allowed (%)  Disallowed (%) | 95.79  4.21  0 | 95.79  4.21  0 | 95.79  4.21  0 | 91.58  8.42  0 |
| EMDB | EMD-56597 | EMD-56600 | EMD-56599 | EMD-56601 |
| PDB | 28LJ | 28LP | 28LO | 28LQ |

Table S2: Cryo-EM data collection, refinement and validation statistics

|  | D252V  Pick  Singlet | D252V  Pick  doublet | ΔG389_I392  PHF | G272V  Pick  singlet | G272V  Pick  doublet |
| --- | --- | --- | --- | --- | --- |
| **Data collection** |  | | | | |
| Microscope | Titan Krios | | | | |
| Voltage (kV) | 300 kV | | | | |
| Detector | Falcon4i | Falcon4i | Falcon4i | Falcon4i | Falcon4i |
| Electron exposure (e–/Å^2^) | 40 | 40 | 40 | 40 | 40 |
| Defocus range (μm) | -1.2 to -2.4 | -1.2 to -2.4 | -1.2 to -2.4 | 1.2 to -2.4 | 1.2 to -2.4 |
| Pixel size (Å) | 0.824 | 0.824 | 1.488 | 0.824 | 0.824 |
| **Data processing** |  |  |  |  |  |
| Box size (pixel) | 384 | 384 | 192 | 384 | 384 |
| Symmetry imposed | C1 | C2 | C1 | C1 | C2 |
| Initial particle images (no.) | 824,712 | 824,712 | 731546 | 941121 | 941121 |
| Final particle images (no.) | 186,885 | 120,538 | 183261 | 303459 | 19556 |
| Map resolution (Å)  FSC threshold 0.143 | 3.0 | 2.7 | 3.0 | 3.9 | 3.1 |
| Helical rise (Å) | 4.86 | 4.85 | 2.43 | 4.849 | 4.83 |
| Helical twist (°) | -0.61 | -0.74 | 179.43 | -0.67 | -0.79 |
| **Refinement** |  |  |  |  |  |
| Model resolution (Å)  FSC threshold 0.5 | 3.7 | 2.8 |  | 5.3 | 3.5 |
| Map sharpening *B* factor (Å^2^) | -47 | -50 |  | -16.9 | -37 |
| Model composition  Non-hydrogen atoms  Protein residues  Ligands | 1764  234  0 | 3528  468  0 |  | 1629  216  0 | 3258  432  0 |
| *B* factors (Å^2^)  Protein | 57.4 | 57.4 |  | 47.4 | 47.4 |
| R.m.s. deviations  Bond lengths (Å)  Bond angles (°) | 0.010  1.927 | 0.010  1.928 |  | 0.010  2.283 | 0.010  2.288 |
| Validation  MolProbity score  Clashscore  Poor rotamers (%) | 0.77  0.00  1.96 | 0.76  0.00  1.72 |  | 1.56  0.00  4.23 | 1.58  0.00  4.50 |
| Ramachandran plot  Favored (%)  Allowed (%)  Disallowed (%) | 97.81  2.19  0 | 97.59  2.41  0 |  | 88.57  10.95  0.48 | 88.57  11.19  0.24 |

|  | S320F  Pick | S320F  Pick  doublet | S320F  Type 2 | S320F  Type 2 doublet |
| --- | --- | --- | --- | --- |
| **Data collection** |  | | | |
| Microscope | Titan Krios | | | |
| Voltage (kV) | 300 kV | | | |
| Detector | Falcon4i | Falcon4i | Falcon4i | Falcon4i |
| Electron exposure (e–/Å^2^) | 40 | 40 | 40 | 40 |
| Defocus range (μm) | 1.2 to -2.4 | 1.2 to -2.4 | -1.2 to -2.4 | -1.2 to -2.4 |
| Pixel size (Å) | 0.824 | 0.824 | 0.824 | 0.824 |
| **Data processing** |  |  |  |  |
| Box size (pixel) | 384 | 384 | 384 | 384 |
| Symmetry imposed | C1 | C2 | C1 | C2 |
| Initial particle images (no.) | 2,140,160 | 2,140,160 | 2,140,160 | 2,140,160 |
| Final particle images (no.) | 135,694 | 84,814 | 238,580 | 48,169 |
| Map resolution (Å)  FSC threshold 0.143 | 2.6 | 2.6 | 2.2 | 2.9 |
| Helical rise (Å) | 4.85 | 4.84 | 4.82 | 4.82 |
| Helical twist (°) | -0.76 | -0.78 | -1.64 | -1.15 |
| **Refinement** |  |  |  |  |
| Model resolution (Å)  FSC threshold 0.5 | 3.1 | 2.8 | 2.7 | 3.2 |
| Map sharpening *B* factor (Å^2^) | -33 | -42 | -35 | -43 |
| Model composition  Non-hydrogen atoms  Protein residues  Ligands | 2115  279  0 | 4175  552  0 | 1770  219  0 | 3408  438  0 |
| *B* factors (Å^2^)  Protein | 59.1 | 58.8 | 96.5 | 96.47 |
| R.m.s. deviations  Bond lengths (Å)  Bond angles (°) | 0.011  2.154 | 0.011  2.008 | 0.010  1.927 | 0.011  1.965 |
| Validation  MolProbity score  Clashscore  Poor rotamers (%) | 1.13  0.00  0.00 | 1.23  0.47  0.21 | 0.5  0.00  0.00 | 0.77  0.00  0.00 |
